# Clinical effect of progressive pulmonary fibrosis on patients with connective tissue disease-associated interstitial lung disease: a single center retrospective cohort study

**DOI:** 10.1007/s10238-023-01212-z

**Published:** 2023-10-13

**Authors:** Ju Kwang Lee, Yura Ahn, Han Na Noh, Sang Min Lee, Bin Yoo, Chang-Keun Lee, Yong-Gil Kim, Seokchan Hong, Soo Min Ahn, Ho Cheol Kim

**Affiliations:** 1 Department of Internal Medicine, Asan Medical Center, University of Ulsan College of Medicine, Seoul, Republic of Korea; 2 Department of Radiology, Asan Medical Center, University of Ulsan College of Medicine, Seoul, Republic of Korea; 3 Division of Rheumatology, Department of Internal Medicine, Asan Medical Center, University of Ulsan College of Medicine, Seoul, Republic of Korea; 4 Department of Pulmonary and Critical Care Medicine, Asan Medical Center, University of Ulsan College of Medicine, 88 Olympic-Ro 43-Gil, Songpa-Gu, Seoul, 05505 Republic of Korea

**Keywords:** Connective tissue disease, Interstitial lung disease, Mortality, Progressive pulmonary fibrosis

## Abstract

The concept of progressive pulmonary fibrosis (PPF) has been introduced to predict the diverse prognosis of interstitial lung disease (ILD). However, the incidence and effect of PPF on outcomes in patients with connective tissue disease-associated interstitial lung disease (CTD-ILD) need to be elucidated. This study reviewed 197 patients with CTD-ILD. Symptomatic worsening, pulmonary function decline, and radiological deterioration were investigated to assess the fulfillment of PPF diagnostic criteria. Clinical outcomes, including mortality, were compared based on the presence or absence of PPF. The median follow-up duration was 17.4 months. The mean age of the patients was 64.0 years, and 60.9% were female. Among the underlying CTDs, rheumatoid arthritis (42.1%), inflammatory myositis (19.8%), and systemic sclerosis (13.2%) were the most common. Of the 197 patients, 37 (18.8%) met the diagnostic criteria for PPF during the follow-up period. Even after adjusting for other significant risk factors, PPF was independently associated with mortality [hazard ratio (HR) 3.856; 95% confidence interval (CI) 1.387–10.715; P = 0.010] and baseline albumin was marginally significantly associated with mortality (HR 0.549; CI 0.298–1.010; P = 0.054). The median survival was also significantly shorter in the PPF group than in the non-PPF group (72.3 ± 12.9 vs. 126.8 ± 15.5 months, *P* < 0.001). Baseline KL-6 ≥ 1000 (U/mL) was a significant risk factor for PPF (HR 2.885; CI 1.165–7.144; *P* = 0.022). In addition to increased mortality, the PPF group had significantly higher rates of respiratory-related hospitalizations, pneumonia, acute exacerbations, and weight loss than the non-PPF group. PPF is a significant prognostic indicator in patients with CTD-ILD. Thus, healthcare professionals should know that patients with CTD-ILD are at risk of PPF.

**Supplementary Information:**

The online version contains supplementary material available at 10.1007/s10238-023-01212-z.

## Background

Connective tissue disease (CTD) is a heterogeneous disease entity including various diseases such as rheumatoid arthritis, systemic sclerosis, inflammatory myositis, systemic lupus erythematosus, and Sjögren’s syndrome. These CTDs can cause multiple symptoms and signs and affect patients’ quality of life and survival [1, 2].

Particularly, interstitial lung disease (ILD) often coexists with CTD, and ILD has a significant effect on the morbidity and mortality of patients with CTD [3, 4]. ILD concurrent with CTD is called connective tissue disease-associated interstitial lung disease (CTD-ILD). The clinical course of CTD-ILD is very diverse, from asymptomatic to fulminant and life-threatening disease [5, 6]. Recently, several guidelines and statements have been suggested for the management of CTD-ILD [7–10]. However, until now, no gold standard has been established for CTD-ILD management.

The concept of progressive pulmonary fibrosis (PPF) has recently been introduced. PPF is defined as pulmonary fibrosis without idiopathic pulmonary fibrosis (IPF) that satisfied at least two of the symptoms, pulmonary function, or radiologic deterioration within the last 1 year [11]. Currently, PPF is being validated for its ability to predict the prognosis of patients with ILD in follow-up studies [12, 13].

According to previous studies, the prevalence of PPF in patients with CTD-ILD ranged from 23 to 38% [14–16]. However, to our knowledge, there is currently no large-scale, well-designed study available to investigate the clinical impacts of PPF in patients with CTD. Therefore, in this study, we investigated the clinical characteristics of patients with CTD who met the definition of PPF and the clinical effect of PPF on the course of CTD-ILD.

## Methods

### Study population

We retrospectively collected data of 197 patients with CTD-ILD from a tertiary referral hospital of 2700 beds in Seoul, South Korea. Patients who were diagnosed with CTD-ILD between April 1, 2007, and October 27, 2022, were included. The medical records of these patients were retrospectively analyzed in January 2023.

CTD was diagnosed by rheumatologists using specific criteria. Systemic sclerosis, rheumatoid arthritis, and systemic lupus erythematosus were diagnosed based on the diagnostic criteria established by the American College of Rheumatology [17–19]. Mixed connective tissue disease was diagnosed using the diagnostic criteria developed by Alarcon-Segovia [20]. Dermatomyositis and polymyositis were diagnosed according to the diagnostic and classification criteria established by Bohan and Peter [21]. Undifferentiated connective tissue disease was defined using classification criteria [22]. Sjögren’s syndrome was diagnosed based on the revised criteria proposed by the American-European Consensus Group [23].

Due to the absence of a single golden standard in the diagnosis of CTD-ILD, a multidisciplinary approach involving rheumatologists, pulmonologists, and radiologists was employed to diagnose CTD-ILD [24]. The diagnostic criteria for acute exacerbation of CTD-ILD were defined as follows, similar to those proposed by Collard et al. [25] for IPF patients: it is characterized by the occurrence or worsening of respiratory distress within the last 1 month in patients diagnosed with CTD-ILD. Additionally, chest computed tomography (CT) scans must reveal new bilateral ground-glass opacities and/or consolidation, and the deterioration cannot be fully explained by conditions such as cardiac failure or fluid overload.

This study was conducted following the Declaration of Helsinki. The Institutional Review Board of Asan Medical Center endorsed this study protocol (IRB no. 2022-1564) The Institutional Review Board waived the need for informed consent because of anonymous clinical data and retrospective study design.

### Clinical data

Clinical and survival data were collected from the medical records and the National Health Insurance of Korea database. We analyzed data from regular follow-up clinic visits or hospitalizations. Spirometry, forced vital capacity (FVC), diffusing capacity of the lung for carbon monoxide (DLco), and total lung capacity (TLC) measurements were reviewed following the guidelines provided by the American Thoracic Society/European Respiratory Society [26–28]. The outcomes were reported as a percentage of the expected normal values. The 6-min walk test (6MWT) was also performed following guidelines from the American Thoracic Society [29].

In addition, high-resolution computed tomography (HRCT) was performed in accordance with standard protocols at full inspiration without contrast enhancement. HRCT images were independently reviewed by two thoracic radiologists (YA and HNN) blinded to the clinical and pathologic information. HRCT images acquired at diagnosis and within a year following diagnosis were reviewed. On HRCT, initial radiologic classification (usual interstitial pneumonia (UIP) vs. non-UIP pattern) was determined and an increased extent of fibrosis was visually assessed with a side-by-side comparison [30]. Disagreement between readers was resolved via a consensus. A questionnaire was not used, but an electronic medical record within a year, to identify the worsening of symptoms and functional capacity.

PPF was diagnosed according to 2022 clinical practice guidelines [11], which defined PPF as meeting a minimum of two of the following three criteria: a decrease of at least 5% in the absolute value of the FVC or a decrease of at least 10% in DLco, worsening of symptoms, and radiological evidence indicating disease progression. To identify death and its dates, we used data from National Insurance Corporation from the diagnosis to January 1, 2023. In-hospital data were used for demographics, comorbidity, baseline laboratory test such as KL-6 and autoantibodies, administration of immunosuppressive agents and steroids, and their side effects.

### Outcome analysis

Patients with CTD-ILD were divided into CTD-ILD groups with PPF and without PPF according to whether they met the diagnostic criteria for PPF, and the differences in mortality between the two groups were analyzed. Factors that affect mortality in patients with CTD-ILD and risk factors of PPF were also investigated. Subgroup analysis was performed on the most prevalent CTDs, rheumatoid arthritis, and inflammatory myositis, to investigate the effect of PPF on mortality differs according to CTD types.

### Statistical analysis

All continuous variables are presented as means ± standard deviations, and categorical variables are presented as percentages. Student’s t-test or Mann–Whitney U-test was used to compare continuous variables, and the chi-square or Fisher’s exact test was employed to compare categorical variables. Cox proportional hazard analyses were performed to calculate the univariate and multivariate hazard ratios (HRs) of mortality according to PPF and disease entity. Cox proportional hazard analyses were also performed to determine risk factors and calculate the univariate and multivariate HRs of PPF according to potential risk factors. The Kaplan–Meier method with a log-rank test was used to estimate the cumulative mortality rates according to disease entity and presence of PPF and compare them. Variables with a *P*-value < 0.1 in the univariate analysis were only included in the multivariate analysis. All statistical tests were two-sided, and a *P*-value of < 0.05 was considered significant. All statistical analyses were performed using IBM SPSS version 24.0 (IBM Corp., Armonk, NY, USA).

## Results

### Patients

**Fig. 1:**
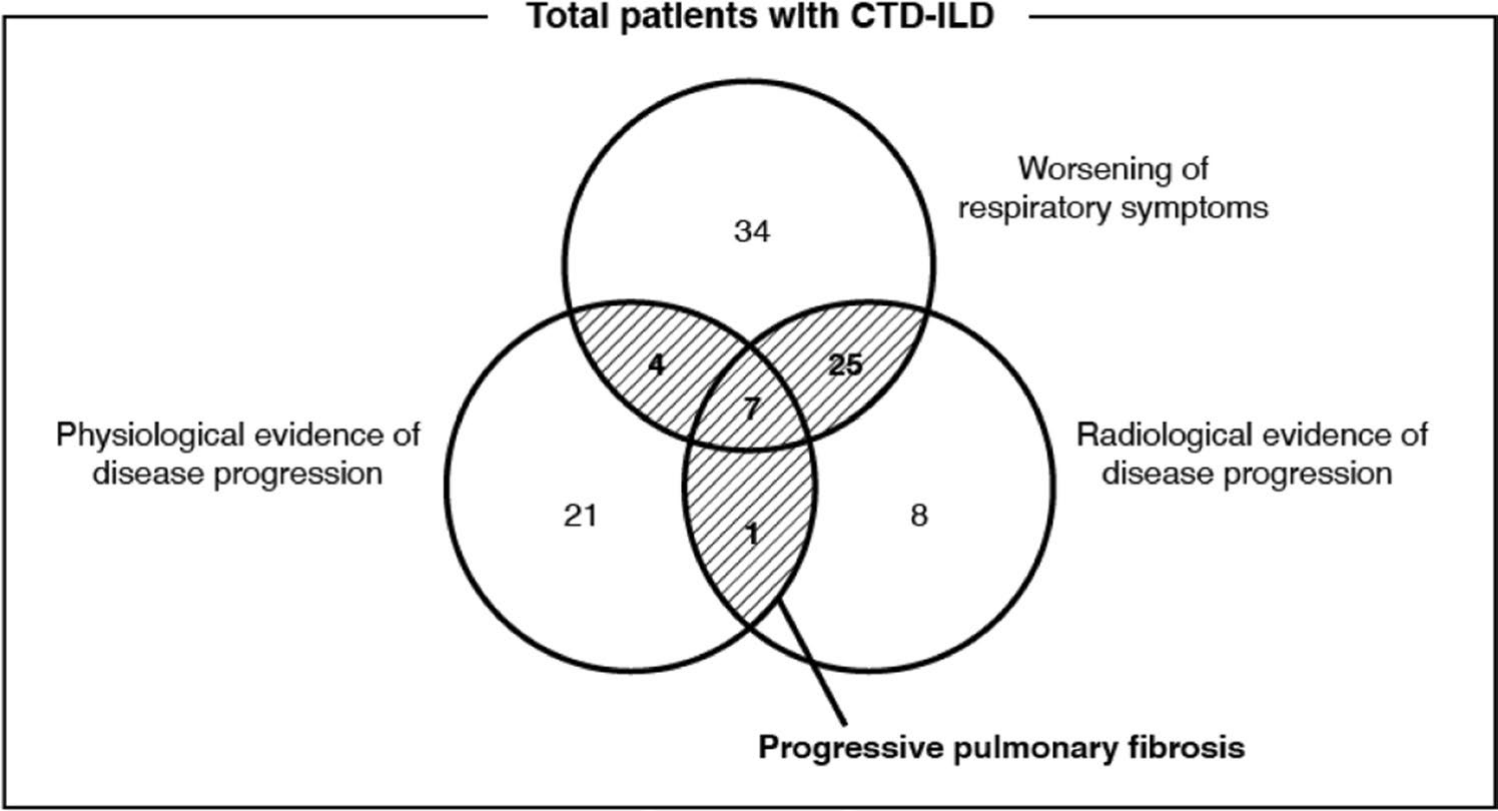
Venn diagram of the satisfaction status of each diagnostic criterion of PPF among 197 patients with connective tissue disease-associated interstitial lung disease

The median follow-up duration was 17.4 [interquartile range (IQR): 7.4–29.4] months. Of the 197 patients with CTD-ILD included in this study, 37 (18.8%) fulfilled the criteria for PPF during the follow-up period. When the 197 patients were categorized based on the three diagnostic criteria for PPF, 70 (35.5%) experienced symptomatic worsening, 41 (20.8%) exhibited radiological worsening, and 33 (16.8%) showed physiological worsening (decline in pulmonary function) (Fig. 1).

The baseline clinical characteristics of the two groups, divided based on the satisfaction of PPF diagnostic criteria, are presented in Table 1. In the PPF group, the baseline Krebs von den Lungen-6 (KL-6) level was significantly higher (median 1055.7, IQR 597.3–2213.5 vs. median 572.0, IQR 354.2–926.6, *P* < 0.001), whereas the albumin level was lower (median 3.0, IQR 2.4–3.7 vs. median 3.6, IQR 3.2–3.8, *P* < 0.001, respectively). Regarding the underlying CTD, the frequency of rheumatoid arthritis was significantly lower, whereas the frequency of inflammatory myositis was higher in the PPF group (27.0% vs. 45.6%, *P* = 0.039; 40.5% vs. 15.0%, *P* < 0.001, respectively). Pulmonary function parameters were also significantly lower in the PPF group (FVC, 60.8 ± 14.9 vs. 73.9 ± 17.4, *P* < 0.001; DLco, 46.1 ± 17.3 vs. 55.6 ± 17.4, *P* = 0.010; TLC, 67.1 ± 12.6 vs. 78.1 ± 15.8, *P* = 0.002). Moreover, the 6-min walking distance (6MWD) was significantly shorter in the PPF group (median 385.0, IQR 280.5–483.0 vs. median 455.0, IQR 360.0–507.5, *P* = 0.042).

**Table 1:** Clinical characteristics of 197 patients with connective tissue disease-associated interstitial lung disease based on progressive pulmonary fibrosis

Characteristics	Total (N = 197)	With progressive pulmonary fibrosis (n = 37)	Without progressive pulmonary fibrosis (n = 160)	*P*-value
Age (years)	64.0 (52.5–69.5)	64.0 (51.0–69.5)	64.0 (53.0–69.8)	0.997
Male sex	77 (39.1)	17 (45.9)	60 (37.5)	0.343
Ever smoker	74 (37.6)	14 (37.8)	60 (37.5)	0.969
Baseline BMI (kg/m^2^, n = 193)	23.1 (20.9–25.5)	23.6(20.1–25.3)	23.1 (21.1–25.6)	0.798
Baseline KL-6 (U/mL, n = 122)	611.9 (395.5–1079.7)	1055.7 (597.3–2213.5)	572.0 (354.2–926.6)	< 0.001
Baseline albumin (g/dL, n = 192)	3.5 (3.1–3.8)	3.0 (2.4–3.7)	3.6 (3.2–3.8)	< 0.001
Radiologic UIP pattern	52 (26.4)	11 (29.7)	41 (25.6)	0.610
Underlying connective tissue disease				
Rheumatoid arthritis	83 (42.1)	10 (27.0)	73 (45.6)	0.039
Inflammatory myositis	39 (19.8)	15 (40.5)	24 (15.0)	< 0.001
Systemic sclerosis	26 (13.2)	3 (8.1)	23 (14.4)	0.423
Systemic lupus erythematosus	21 (10.7)	4 (10.8)	17 (10.6)	> 0.999
Sjögren’s syndrome	19 (9.6)	4 (10.8)	15 (9.4)	0.761
Undifferentiated connective tissue disease	5 (2.5)	1 (2.7)	4 (2.5)	> 0.999
Mixed connective tissue disease	4 (2.0)	0 (0.0)	4 (2.5)	> 0.999
Baseline pulmonary function test				
FVC (% predicted, n = 176)	71.7 ± 17.7	60.8 ± 14.9	73.9 ± 17.4	< 0.001
DLco (% predicted, n = 169)	54.1 ± 17.7	46.1 ± 17.3	55.6 ± 17.4	0.010
TLC (% predicted, n = 135)	76.3 ± 15.9	67.1 ± 12.6	78.1 ± 15.8	0.002
6MWD (meter, n = 147)	450.0 (360.0–505.0)	385.0 (280.5–483.0)	455.0 (360.0–507.5)	0.042
6MWT, lowest SpO2 (%, n = 147)	94.0 (90.0–97.0)	91.0 (85.5–97.0)	95.0 (91.0–97.0)	0.097

Data are presented as the mean ± standard deviation, median (interquartile range), or number (percentage)

*UIP* usual interstitial pneumonia, *6MWD* 6-min walk test distance, *6MWT* 6-min walk test, *DLco* diffusing capacity of the lung for carbon monoxide, *FVC* forced vital capacity, *SpO*_*2*_ peripheral oxygen saturation, *TLC* total lung capacity

When comparing baseline autoantibodies between the PPF group and the non-PPF group, the PPF group showed significantly lower frequencies of high ANA (Anti-nuclear antibody) titer (≥ 320:1) and anti-CCP (cyclic citrullinated peptide) antibody positivity (21.6% vs. 38.8%, *P* = 0.050 and 36.4% vs. 56.8%, *P* = 0.038, respectively, Additional file1: Supplementary Table 1).

### Factors affecting mortality

During follow-up, 38 (19.3%) patients died. Table 2 presents the results of the Cox proportional hazard analysis for the risk factors of mortality. In the univariate analysis, several factors significantly correlated with mortality, including age [HR 1.039; 95% confidence interval (CI) 1.008–1.071; *P* = 0.014], male sex (HR 2.194; CI 1.150–4.187; *P* = 0.017), baseline KL-6 ≥ 1000 (U/mL) (HR 2.818; CI 1.205–6.592; *P* = 0.017), baseline albumin (HR 0.306; CI 0.221–0.424; *P* < 0.001), baseline radiologic UIP pattern (HR 2.528; CI 1.330–4.803; *P* = 0.005), PPF (HR 4.820; CI 2.539–9.152; *P* < 0.001), DLco (HR 0.959; CI 0.936–0.981; *P* < 0.001), 6MWD (HR 0.996; CI 0.992–0.999; *P* = 0.010), and 6MWT and lowest SpO2 (HR 0.855; CI 0.791–0.924; *P* < 0.001).

**Table 2:** Cox proportional hazard analysis of risk factors for mortality in 197 patients with connective tissue disease-associated interstitial lung disease

Characteristics	Hazard ratio	95% Confidence interval	*P*-value
*Univariate analysis*			
Age (years)	1.039	1.008–1.071	0.014
Male sex	2.194	1.150–4.187	0.017
Ever smoker	1.166	0.611–2.224	0.642
Baseline BMI (kg/m^2^, n = 193)	0.987	0.906–1.075	0.761
Baseline KL-6 ≥ 1000 (U/mL, n = 122)	2.818	1.205–6.592	0.017
Baseline albumin (g/dL, n = 192)	0.306	0.221–0.424	< 0.001
Radiologic UIP pattern	2.528	1.330–4.803	0.005
Progressive pulmonary fibrosis	4.820	2.539–9.152	< 0.001
Pulmonary function test			
FVC (% predicted, n = 176)	0.984	0.963–1.005	0.133
DLco (% predicted, n = 169)	0.959	0.936–0.981	< 0.001
TLC (% predicted, n = 135)	0.968	0.937–1.001	0.055
6MWD (meter, n = 147)	0.996	0.992–0.999	0.010
6MWT, lowest SpO2 (%, n = 147)	0.855	0.791–0.924	< 0.001
*Multivariate analysis*			
Age (years)	1.044	0.976–1.115	0.208
Male sex	1.737	0.577–5.235	0.326
Baseline KL-6 ≥ 1000 (U/mL)	2.453	0.694–8.675	0.164
Baseline Albumin (g/dL)	0.549	0.298–1.010	0.054
UIP pattern	2.705	0.799–9.158	0.110
Progressive pulmonary fibrosis	3.856	1.387–10.715	0.010
Pulmonary function test			
DLco (% predicted)	0.979	0.946–1.012	0.210

6MWD (r = 0.996, *P* = 0.010), 6MWT, and lowest SpO2 (r = 0.855, *P* < 0.001) were not included in the multivariate analysis because of the high proportion of the missing data

*UIP* usual interstitial pneumonia, *6MWD* 6-min walk test distance, *6MWT* 6-min walk test, *DLco* diffusing capacity of the lung for carbon monoxide, *FVC* forced vital capacity, *SpO*_*2*_, peripheral oxygen saturation, *TLC* total lung capacity

**Fig. 2:**
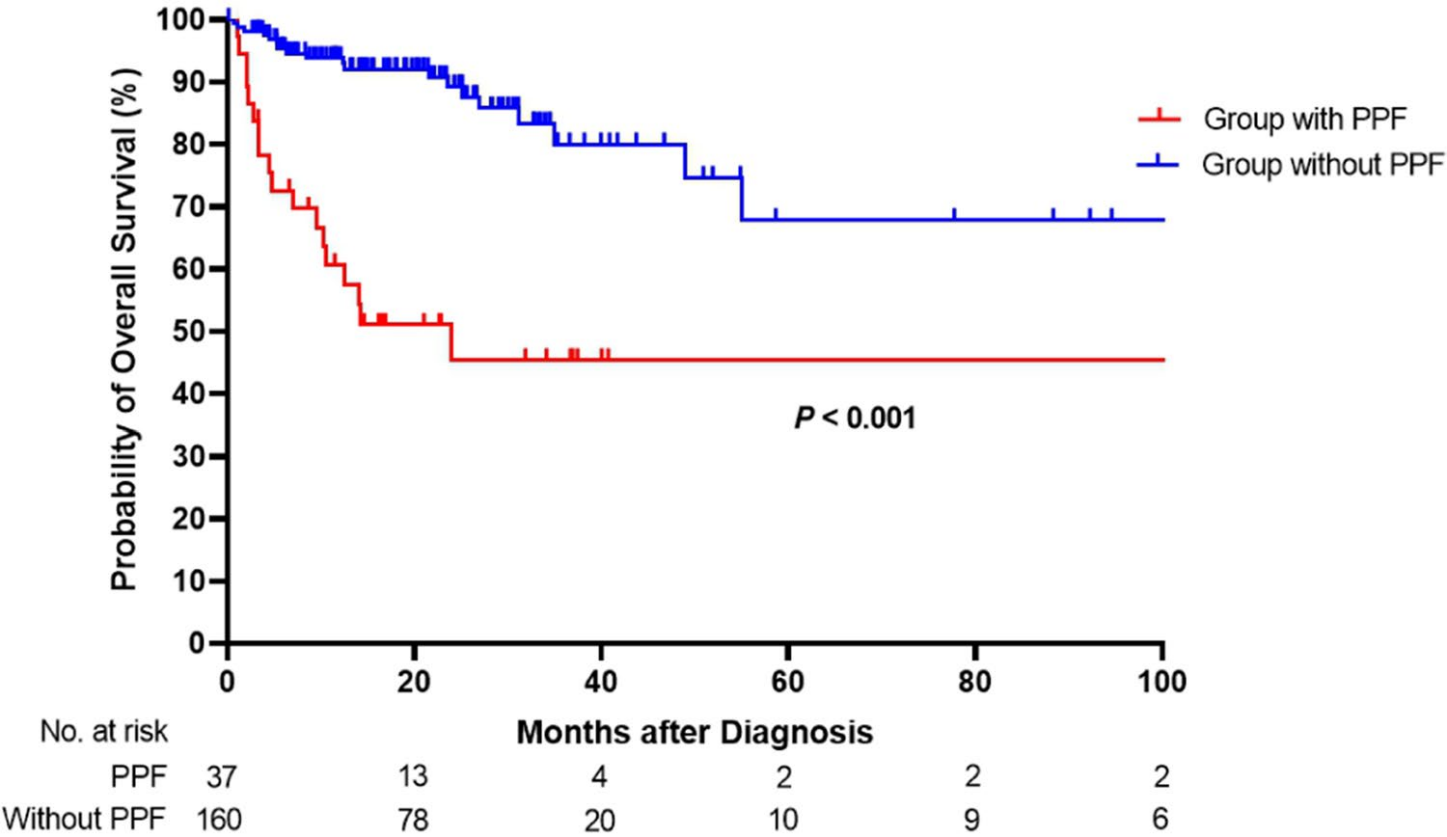
Kaplan–Meier plot of the cumulative mortality rate based on the diagnosis of progressive pulmonary fibrosis in patients with connective tissue disease-associated interstitial lung disease

In the multivariate analysis, only PPF was independently associated with mortality (HR 3.856; CI 1.387–10.715; *P* = 0.010), after adjusting other risk factors, such as age (HR 1.044; *P* = 0.208), male sex (HR 1.737; *P* = 0.326), baseline KL-6 ≥ 1000U/mL (HR 2.453; *P* = 0.164), baseline albumin (HR 0.549; *P* = 0.054), radiologic UIP pattern (HR 2.075; *P* = 0.110), and baseline DLco (HR 0.979; *P* = 0.210). Figure 2 displays the Kaplan–Meier curves divided into two groups based on whether patients with CTD-ILD met the definition of PPF. The median survival was significantly shorter in the PPF group than in the non-PPF group (72.3 ± 12.9 vs. 126.8 ± 15.5 months, *P* < 0.001).

### Survival analysis in subgroups

Survival analyses were conducted for the two most frequent CTDs, namely, rheumatoid arthritis and inflammatory myositis. Among the 83 patients with rheumatoid arthritis, a significant difference was found in the survival curves between the PPF and non-PPF groups (*P* = 0.001, Additional file 2: Figure S1-A). Similarly, among the 39 patients with inflammatory myositis, significant differences were observed in the survival curves between the two groups (*P* = 0.003, Additional file 2: Figure S1-B).

### Risk factors of PPF

In a Cox proportional hazard analysis conducted on 197 patients with CTD-ILD, the univariate analysis revealed several significant risk factors for PPF. These included baseline KL-6 ≥ 1000 (U/mL) (HR 4.371; CI 2.037–9.376; *P* < 0.001), baseline albumin levels (HR 0.422; CI 0.292–0.610; *P* < 0.001), baseline pulmonary function measurements of FVC (HR 0.963; CI 0.945–0.982; *P* < 0.001) DLco, (HR 0.963; CI 0.941–0.986; *P* = 0.002), and 6MWD (HR 0.997; CI 0.994–1.000; *P* = 0.042). In the multivariate analysis, the baseline KL-6 ≥ 1000 (U/mL) was statistically significant as a risk factor for PPF (HR 2.885; CI 1.165–7.144; *P* = 0.022), and the baseline lower FVC was marginally associated with PPF (HR 0.963; CI 0.927–1.001; *P* = 0.056) (Table 3).

**Table 3:** Cox proportional hazard analysis of risk factors for progressive pulmonary fibrosis in 197 patients with connective tissue disease-associated interstitial lung disease

Characteristics	Odds ratio	95% Confidence interval	*P*-value
*Univariate analysis*			
Age (years)	1.008	0.981–1.036	0.563
Male sex	1.377	0.721–2.629	0.332
Ever smoker	0.979	0.504–1.903	0.950
Baseline BMI (kg/m^2^, n = 193)	0.972	0.887–1.066	0.546
Baseline KL-6 ≥ 1000 (U/mL, n = 122)	4.371	2.037–9.376	< 0.001
Baseline Albumin (g/dL, n = 192)	0.422	0.292–0.610	< 0.001
Radiologic UIP pattern	1.336	0.659–2.709	0.422
Baseline pulmonary function test			
FVC (% predicted, n = 176)	0.963	0.945–0.982	< 0.001
DLco (% predicted, n = 169)	0.963	0.941–0.986	0.002
TLC (% predicted, n = 135)	0.949	0.921–0.979	0.001
6MWD (meter, n = 147)	0.997	0.994–1.000	0.042
6MWT, lowest SpO2 (%, n = 147)	1.002	0.997–1.008	0.398
*Multivariate analysis*			
Baseline KL-6 ≥ 1000 (U/mL)	2.885	1.165–7.144	0.022
Baseline Albumin (g/dL)	0.608	0.329–1.124	0.113
Baseline pulmonary function test			
FVC (% predicted)	0.963	0.927–1.001	0.056
DLco (% predicted)	0.998	0.961–1.038	0.938

TLC (r = 0.949, *P* = 0.001) was excluded in the multivariate analysis because of its close correlation with the FVC. 6MWD (r = 0.997, *P* = 0.042) was not included in the multivariate analysis because of the high proportion of the missing data

*UIP* usual interstitial pneumonia, *6MWD* 6-min walk test distance, *6MWT* 6-min walk test, *DLco* diffusing capacity of the lung for carbon monoxide, *FVC* forced vital capacity, *SpO*_*2*_ peripheral oxygen saturation, *TLC* total lung capacity

### Treatment and clinical outcomes

As shown in Table 4, among the 197 patients with CTD-ILD, 163 (82.7%) were on steroid therapy. A significantly higher proportion of patients with PPF was on steroid therapy than patients without PPF (35 of 37, 94.6% vs. 128 of 160, 80.0%, *P* = 0.034). Furthermore, the maximal dose of steroids was significantly higher in the PPF group (median 57.5 mg, IQR 30.0–75.0) than in the non-PPF group (median 30.0 mg, IQR 7.5–43.8, *P* < 0.001), and the PPF group had significantly shorter duration of steroid treatment (median 9.0 months, IQR 4.0–14.0) than the non-PPF group (median 14.0 months, IQR 6.0–36.8, *P* = 0.008).

**Table 4:** Treatment and clinical outcomes of 197 patients with connective tissue disease-associated interstitial lung disease based on progressive pulmonary fibrosis

Characteristics	Total (N = 197)	With progressive pulmonary fibrosis (n = 37)	Without progressive pulmonary fibrosis (n = 160)	*P*-value
*Treatment*				
Usage of steroid	163 (82.7)	35 (94.6)	128 (80.0)	0.034
Maximal dose of steroid(prednisolone, mg)	30.0 (7.5–50.0)	57.5 (30.0–75.0)	30.0 (7.5–43.8)	< 0.001
Duration of steroid treatment (months)	12.0 (6.0–31.0)	9.0 (4.0–14.0)	14.0 (6.0–36.8)	0.008
Usage of immunosuppressant	93 (47.2)	27 (73.0)	66 (41.3)	< 0.001
Mycophenolate mofetil	51 (25.9)	22 (59.5)	29 (18.1)	< 0.001
Azathioprine	41 (20.8)	7 (18.9)	34 (21.3)	0.753
Cyclosporine	9 (4.6)	2 (5.4)	7 (4.4)	0.678
Cyclophosphamide	1 (0.5)	0 (0.0)	1 (0.6)	> 0.999
Tocilizumab	11 (5.6)	3 (8.1)	8 (5.0)	0.436
*Clinical outcomes*				
Respiratory hospitalization	62 (31.5)	30 (81.1)	32 (20.0)	< 0.001
Pneumonia	55 (27.9)	29 (78.4)	26 (16.3)	< 0.001
Acute exacerbation	47 (23.9)	24 (64.9)	23 (14.4)	< 0.001
Significant weight loss	25 (18.7)	8 (36.4)	17 (15.2)	0.033
Death	38 (19.3)	18 (48.6)	20 (12.5)	< 0.001

Data are presented as the median (interquartile range), or number (percentage)

Significant weight loss was defined as a weight loss of ≥ 5% over 1 year after diagnosis

Among all patients with CTD-ILD, 47.2% (93 of 197) received immunosuppressant therapy. Immunosuppressive agents were significantly more commonly administered to the PPF group than to the non-PPF group (73.0%, 27 of 37 vs. 41.3%, 66 of 160, *P* < 0.001). Mycophenolate mofetil was the most commonly used immunosuppressant, followed by azathioprine, cyclosporine, cyclophosphamide, and tocilizumab. The use of mycophenolate mofetil was significantly higher in the PPF group than in the non-PPF group (59.5% vs. 18.1%, *P* < 0.001).

Regarding clinical outcomes, of the total 197 patients, 38 (19.3%) died. During the follow-up period, significantly more deaths were observed in the PPF group than in the non-PPF group (18 of 37, 48.6% vs. 20 of 160, 12.5%, *P* < 0.001). Comparing other outcomes, respiratory-related hospitalizations were significantly higher in the PPF group than in the non-PPF group (81.1% vs. 20.0%, *P* < 0.001). Pneumonia and acute exacerbation were also significantly more prevalent in the PPF group than in the non-PPF group (78.4% vs. 16.3%, *P* < 0.001 and 64.9% vs. 14.4%, *P* < 0.001, respectively). Significant weight loss defined as a decrease of ≥ 5% in body weight was also significantly higher in the PPF group than in the non-PPF group (36.4% vs. 15.2%, *P* = 0.033, Table 4).

## Discussion

This study investigated the frequency and clinical outcomes of PPF in patients with CTD-ILD. Approximately one-fifth of patients (18.8% of study populations) met the PPF criteria, and PPF was independently associated with mortality (HR 3.856; CI 1.387–10.715; *P* = 0.010). In addition, despite more treatment, the PPF group showed worse survival than the non-PPF group.

According to previous studies, the prevalence of PPF in patients with CTD-ILD ranged from 23 to 38% [14–16]. In the present study, 37 (18.8%) patients met the diagnostic criteria of PPF, which is relatively lower than those in previous studies. These results might be attributed to the difference in the study design. Lee et al. [14] analyzed 107 patients with CTD and identified PPF in 38.3% of these patients. In addition, Chiu et al. [15] reported that 53 (23%) of their 230 patients with CTD-ILD had a PPF with median follow-up of 6 years. The lower prevalence of PPF in the present study than in previous studies may be attributed to several factors. First, the shorter follow-up period in the present study than in previous studies might have contributed to the lower observed prevalence (median follow-up duration: 17.4, IQR; 7.4–29.4 months) when compared with the median follow-up duration of 49.9 ± 36.5 months reported by Lee et al. and of 6 (IQR 6–9) years by Chiu et al. [14, 15]. In addition, the application of stricter criteria for radiologic deterioration, assessed by two radiologists, could have resulted in a more stringent evaluation. Despite potential variations in the criteria used to define PPF, a relatively large-scale study involving 1,749 patients demonstrated that 16.6% (42 of 253 individuals) of patients with CTD exhibited a progressive fibrosing ILD phenotype [31]. To determine the exact proportion of PPF in patients with CTD-ILD, further follow-up studies with a larger sample size are necessary.

In the present study, PPF was independently associated with mortality even after adjusting for other factors, such as baseline albumin level and baseline radiologic pattern, and our finding supports previous reports [12, 13, 16, 32]. Previously, several staging systems using baseline characteristics were suggested for predicting prognosis in patients with CTD-ILD, particularly in systemic sclerosis and rheumatoid arthritis [33–35]. To the best of our knowledge, a few studies have clinically compared the PPF and non-PPF groups only among patients with CTD. Khor et al. [13] analyzed 753 patients with non-IPF fibrotic ILD (including 372 patients with CTD) and reported that PPF was independently associated with increased mortality and lung transplantation (HR 2.08). In addition, Pugashetti et al. [12] enrolled 1341 patients (including 516 patients with CTD) and showed that ≥ 10% relative FVC decline was related to reduced survival in the patients with non-IPF ILD, and additional PPF criteria were associated with reduced transplant-free survival. The present study indicates that important prognostic factors in CTD-ILD include not only initial characteristics such as baseline lung function, CT pattern, and ILD extent but also the clinical course including changes in lung function, symptoms, and radiological findings.

Interestingly, this study showed that despite the higher usage of treatments such as steroids and immunosuppressive agents in the PPF group than in the non-PPF group, they had a worse prognosis. Although several drugs have beneficial effects on CTD-ILD, no gold standard has been established for the management of patients with CTD-ILD [36–38]. Specifically, in patients with a progressive course, such as those in the PPF group, more studies are needed to evaluate the effectiveness of treatments, including antifibrotic agents [39].

It is challenging to directly compare the group that received steroid and/or immunosuppressive therapy with the group that did not, as more than 80% of the overall patients underwent such treatment. Additionally, analyzing the cause of death is also difficult, as in more than half of the deceased patients (20 of 38), the exact cause of death remains unknown. Nonetheless, as shown in Table 4, individuals with PPF encountered a greater frequency of acute exacerbations, hospitalizations due to respiratory issues, and notably, a heightened susceptibility to pneumonia. In light of these findings, it is reasonable to infer that the unfavorable clinical outcome in PPF patients could have been impacted not solely by the severity of the underlying ILD but also by the presence of infections.

Although the criteria for defining the progressive fibrosing phenotype may vary across studies, previous studies have proposed several clinical variables in predicting this phenotype [40, 41]. Hambly et al. [40] analyzed 2746 patients with fibrotic ILD and identified age, male sex, gastroesophageal reflux disease, and impaired lung function as indicators of progression. Among studies on CTD, a retrospective study found male sex and South Asian ethnicity independently predicted a decline in FVC, whereas male sex, smoking history, and systemic sclerosis as the specific CTD type were associated with a decline in DLco [41]. In the present study, the baseline KL-6 ≥ 1000 (U/mL) was identified as a statistically significant risk factor for PPF (HR 2.885; CI 1.165–7.144; *P* = 0.022), and the baseline FVC was borderline significantly associated with PPF (HR 0.963; CI 0.927–1.001; *P* = 0.056). In a previous study of anti-synthetase syndrome-associated ILD with 72 participants, the baseline KL-6 was reported as a significant risk factor for PPF [42]. Although research on the prevalence and risk factors of ILD occurrence in patients with CTD is relatively scarce [41], risk factors for disease progression in CTD-ILD are not yet well elucidated; thus, further research is needed.

Another intriguing finding is that there was a significant relationship between baseline albumin levels and mortality as well as PPF in the univariate analysis (HR 0.306; CI 0.221–0.424; *P* < 0.001, HR 0.422; CI 0.292–0.610; *P* < 0.001, respectively), even though it was not significant in the multivariate analysis (HR 0.549; CI 0.298–1.010; *P* = 0.054, HR 0.608; CI 0.329–1.124; *P* = 0.113, respectively). Previous studies report that low albumin levels are associated with increased mortality in various respiratory diseases, possibly reflecting poor nutritional status [24, 43]. Additionally, while the exact mechanism is not well understood, there has been recent research indicating that low albumin levels predict PPF in Sjögren's syndrome-related interstitial pneumonia patients, probably reflecting ongoing inflammation [44]. Although baseline albumin levels were not an independent predictor of PPF in this study's multivariate analysis, further research on this topic is needed in the future.

Generally, a UIP pattern on imaging has been reported as a hallmark of pulmonary fibrosis associated with poor prognosis [45, 46]. In our current study, while the baseline radiologic UIP pattern showed a significant association with mortality in univariate analysis (HR 2.528; CI 1.330–4.803; *P* = 0.005), it only had a borderline significant relationship with mortality in multivariate analysis (HR 2.705; CI 0.799–9.158; *P* = 0.110, Table 2). However, the radiologic UIP pattern did not have a significant association with the development of PPF (HR 1.336; CI 0.659–2.709; *P* = 0.422, Table 3). Therefore, we believe that while baseline image patterns might be important in patients with CTD, factors related to clinical course, like PPF, appear to be more clinically meaningful in these patients.

The limitations of this study include its retrospective, single-center design. In addition, a selection bias may have occurred because the study excluded individuals with short follow-up. Furthermore, standardized methods such as questionnaires were not used to assess the worsening of symptoms. In addition, this retrospective study was unable to adhere to a well-designed protocol regarding the intervals of pulmonary function tests and CT. In South Korea, the antifibrotic agent including nintedanib is not covered by healthcare insurance. Therefore, owing to its high cost, the majority of patients with PPF had not received nintedanib; thus, the effectiveness of antifibrotic agents could not be analyzed. However, despite the retrospective design of this study, the review of CT scans by two radiologists for the diagnosis of PPF is considered an advantage.

## Conclusions

In conclusion, our findings indicate that PPF could be a significant prognostic indicator in individuals with CTD-ILD. As a result, healthcare professionals should be aware that patients with CTD-ILD are at risk of developing PPF.

### Supplementary Information

Below is the link to the electronic supplementary material.

**Additional file 1: Supplementary TABLE 1.** Baseline autoantibodies of 197 patients with connective tissue disease-associated interstitial lung disease based on progressive pulmonary fibrosis

**Additional file 2: Figure S1.** Kaplan–Meier plot of the cumulative mortality rate based on the diagnosis of progressive pulmonary fibrosis in patients with rheumatoid arthritis-related interstitial lung disease (ILD) (A) and patients with inflammatory myositis-related ILD (B).

## Abbreviations

ANA: Anti-nuclear antibody
CCP: Cyclic citrullinated peptide
CI: 95% Confidence interval
CT: Computed tomography
CTD: Connective tissue disease
CTD-ILD: Connective tissue disease-associated interstitial lung disease
DLco: Diffusing capacity of the lung for carbon monoxide
FVC: Forced vital capacity
HR: Hazard ratio
HRCT: High-resolution computed tomography
ILD: Interstitial lung disease
IPF: Idiopathic pulmonary fibrosis
IQR: Interquartile range
UIP: Usual interstitial pneumonia
KL-6: Krebs von den Lungen-6
PPF: Progressive pulmonary fibrosis
SpO2: Peripheral oxygen saturation
TLC: Total lung capacity
6MWD: 6-Minute walk test distance
6MWT: 6-Minute walk test

## Declarations

### Availability of data and materials

The datasets utilized and/or examined in the present study can be obtained from the corresponding author upon a reasonable request.
