# Supplementary material for: Clinical effect of progressive pulmonary fibrosis on patients with connective tissue disease-associated interstitial lung disease: a single center retrospective cohort study
Source: Clin Exp Med. 2023 Oct 13;23(8):4797–807. doi: 10.1007/s10238-023-01212-z (PMC10725328; doi:10.1007/s10238-023-01212-z)
Supplement: Supplementary file 1 — Additional file 1: Supplementary TABLE 1. Baseline autoantibodies of 197 patients with connective tissue disease-associated interstitial lung disease based on progressive pulmonary fibrosis [file 10238_2023_1212_MOESM1_ESM.docx]

**Supplementary TABLE 1** Baseline autoantibodies of 197 patients with connective tissue disease-associated interstitial lung disease based on progressive pulmonary fibrosis

| Characteristics | Total (N = 197) | With  progressive pulmonary fibrosis (n = 37) | Without  progressive pulmonary fibrosis  (n = 160) | *P*-value |
| --- | --- | --- | --- | --- |
| ANA titer ≥ 320:1 (n = 189) | 67 (35.4) | 8 (21.6) | 59 (38.8) | 0.050 |
| Rheumatoid factor ≥ 60 IU/mL (n = 161) | 63 (39.1) | 10 (30.3) | 53 (41.4) | 0.244 |
| Anti-CCP Ab (n = 151) | 79 (52.3) | 12 (36.4) | 67 (56.8) | 0.038 |
| MPO ANCA (n = 120) | 6(5.0) | 2 (7.1) | 4 (4.3) | 0.623 |
| Anti-RNP Ab (n = 123) | 12 (9.8) | 0 (0.0) | 12 (11.9) | 0.122 |
| Anti-Sm Ab (n = 123) | 5 (4.1) | 0 (0.0) | 5 (5.0) | 0.584 |
| Anti-SS-A Ab (n = 134) | 55 (41.0) | 13 (46.4) | 42 (39.6) | 0.515 |
| Anti-SS-B Ab (n = 134) | 10 (7.5) | 2 (7.1) | 8 (7.5) | >0.999 |
| Anti-Scl-70 Ab (n = 124) | 13 (10.5) | 0 (0.0) | 13 (12.9) | 0.124 |
| Anti-Jo-1 Ab (n = 97) | 5 (5.2) | 1 (3.6) | 4 (5.8) | >0.999 |
| Anti-ds DNA Ab (n = 116) | 18 (15.5) | 1 (5.0) | 17 (17.7) | 0.193 |

Data are presented as the number (percentage).

ANA, anti-nuclear antibodies; CCP, cyclic citrullinated peptide; MPO ANCA, myeloperoxidase Anti-Neutrophil Cytoplasmic Antibody; RNP, ribonucleoprotein; Sm, Smith; SS-A, Sjögren's-syndrome-related antigen A; SS-B, Sjögren's-syndrome-related antigen B.
