# Supplementary figures and images for: Clinical effect of progressive pulmonary fibrosis on patients with connective tissue disease-associated interstitial lung disease: a single center retrospective cohort study
Source: Clin Exp Med. 2023 Oct 13;23(8):4797–807. doi: 10.1007/s10238-023-01212-z (PMC10725328; doi:10.1007/s10238-023-01212-z)

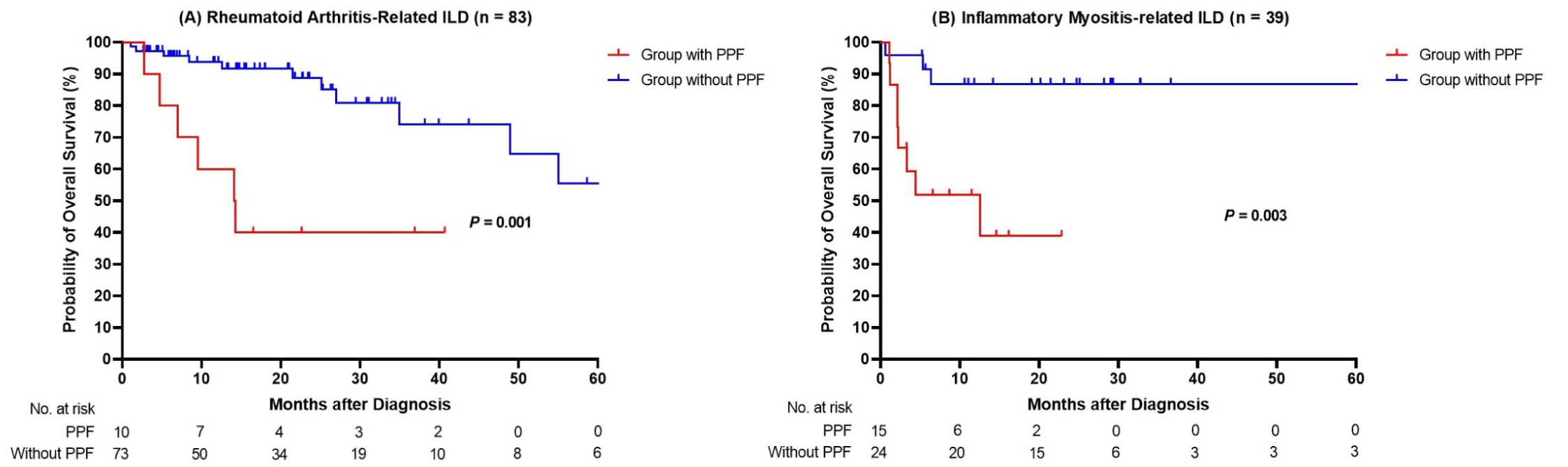

Supplement: Supplementary file 2 — Additional file 2: Figure S1. Kaplan–Meier plot of the cumulative mortality rate based on the diagnosis of progressive pulmonary fibrosis in patients with rheumatoid arthritis-related interstitial lung disease (ILD) (A) and patients with inflammatory myositis-related ILD (B). [file 10238_2023_1212_MOESM2_ESM.jpg]
